# A Case Report of I-Cell Disease (Mucolipidosis Type II) With a Novel Placental-Focused Diagnostic Approach

**DOI:** 10.1177/10935266261440594

**Published:** 2026-04-20

**Authors:** Nicolas LaScala, Laura Rose, Jennifer Sanchez, Jennifer N. Chousal, Sarah Murray, Mana Parast, Betty Pham, Omonigho Aisagbonhi

**Affiliations:** 1 Department of Pathology, University of California San Diego, USA; 2 Department of Pediatrics, University of California San Diego, USA; 3 Division of Neonatology, Department of Pediatrics, University of California, San Diego, USA; 4 Division of Neonatology, Department of Pediatrics, Rady Children’s Hospital, San Diego, CA, USA

**Keywords:** placenta, I-cell disease, lysosomal storage disorders, mucolipidosis type II

## Abstract

Inclusion cell disease (I-cell disease/Mucolipiodsis Type II) is an autosomal recessive lysosomal storage disorder that results in accumulation of substrates (cholesterol, phospholipids, and glycosaminoglycans) in various tissues. The clinical presentation of I-cell disease includes hepatomegaly, splenomegaly, cardiomegaly, upper respiratory infections, skeletal deformities, developmental delay, abnormal facies, and failure to thrive. We describe a case of I-cell disease in a neonate admitted to the Neonatal Intensive Care Unit for respiratory distress with initial suspicion of infection. Placental histopathological findings included syncytiotrophoblast vacuolization and positive lipid vacuoles highlighted with the special stain, Oil Red-O. Ultra-rapid whole genome sequencing from buccal swab identified compound heterozygous *GNPTAB* variants consistent with I-cell disease. Whole exome sequencing of placental tissue showed concordant pathogenic variants. To our knowledge, this is the first report of Oil Red-O staining of intracytoplasmic lipids within syncytiotrophoblast and successfully identifying variants by sequencing placental DNA in the setting of I-cell disease.

## Introduction

Inclusion cell disease (I-cell disease/Mucolipiodsis Type II) is a lysosomal storage disorder and a very rare disease (incidence 1:400 000). It is caused by homozygous or compound heterozygous pathological variants in the *GNPTAB* gene, inherited in an autosomal recessive pattern.^1-4^ Loss of function leads to deficient uridine diphosphate-N-acetylglucosamine:lyosomal enzyme N-acetylglucosamine-1-phosphotransferase (G1cNAc-phosphotransferase) and defective lysosomal trafficking of lysosomal hydrolases from the trans-Golgi network to the lysosome due to lack of tagging with a mannose-6-phosphate. This leads to lysosomal hydrolases being secreted from the cell and accumulation of substrates (cholesterol, phospholipids, and glycosaminoglycans) in various tissues.^2,3^ The clinical presentation of I-cell disease in neonates includes hepatomegaly, splenomegaly, cardiomegaly, upper respiratory infections, skeletal deformities, developmental delay, abnormal facies, and failure to thrive.^4^ Clinical diagnosis is achieved by direct measurement of G1cNAc-phosphotransferase, enzyme assays, biochemical assays, and genetic studies.^1-4.^

Strong clinical suspicion is needed to initiate testing, and neonatal clinical features often overlap with other inherited syndromes or acute illnesses. From a pathologic perspective, disease is present at birth in both the fetus and the placenta, and examination of the placenta shows edematous appearing chorionic villi with loose reticulated mesenchyme, vacuolization of mesenchymal cells and foamy appearing syncytiotrophoblasts with vacuolization.^5-8^ We report a case of I-cell disease in a neonate with characteristic placenta findings, including positive Oil Red-O stain in syncytiotrophoblast intracytoplasmic lipids. To our knowledge, this is the first report with positive staining of intracytoplasmic lipid vacuoles on fresh frozen placenta with Oil Red-O and successfully sequencing variants in the placenta. Our case highlights the utility of placenta examination in evaluation of infants admitted to the neonatal intensive care unit (NICU).

## Case Report

A male infant was born at 40 weeks and 1 day via repeat cesarean section to a 25-year-old G3P2 mother with a history of genital herpes simplex virus, on acyclovir with negative sterile speculum exam. Prenatal labs were unremarkable. Care for this pregnancy began at an outside institution. The mother of the patient presented to our institution late in the pregnancy. Anatomy scan was performed at 36w6d which limited the imaging to detect fetal abnormalities. Additionally, low fetal positioning in the pelvis limited imaging. Notable findings in the anatomy scan included: 22nd percentile for gestational age, lagging head measurements with the circumference measuring at 32 weeks in size (3rd percentile) but not so small to suggest microcephaly with normal intracranial anatomy, and HC:AC ratio borderline low (0.91).

Meconium-stained fluid and nuchal cord were noted at delivery. Apgar scores were 8 and 8 at 1 and 5 minutes respectively. He subsequently developed increased work of breathing requiring continuous positive airway pressure. He was admitted to the NICU for respiratory distress and concern for sepsis. His physical exam was notable for microcephaly, coarse facial features, smooth philtrum, hyperplasia of the gingiva and alveolar ridges, depressed nasal bridge, left talipes equinovares, abnormal left ankle curvature, and diffuse blueberry muffin rash on his face, trunk, arms, and legs.

He was treated with empiric antibiotics for 36 hours. Blood cultures were negative. He received acyclovir for 4 days until HSV testing returned negative. Initial hematological laboratory findings were significant for thrombocytopenia (platelets 39 000/mm^3^). The patient’s labs for TORCH infections (HSV 1/2 DNA, Varicella IgM/IgG, Rubella IgM, Toxoplasma IgM/IgG, urine CMV, and RPR) were all negative. A chest radiograph showed cardiomegaly, hyperexpanded lungs, diffusely abnormal osseous structures with periosteal reaction, subacute bilateral rib fractures, and clavicular irregularities. Skeletal survey showed diffuse diaphyseal cloaking with diffuse periosteal reaction, widened diaphysis, medially angulated left distal tibia and fibula, and irregular fragmentation similar to rickets (Figure 1). Head and abdominal ultrasound were within normal limits. An echocardiogram showed dilated cardiomyopathy without valve thickening, a small atrial septal defect, and a patent ductus arteriosus with left-to-right shunting.

**Figure 1.**
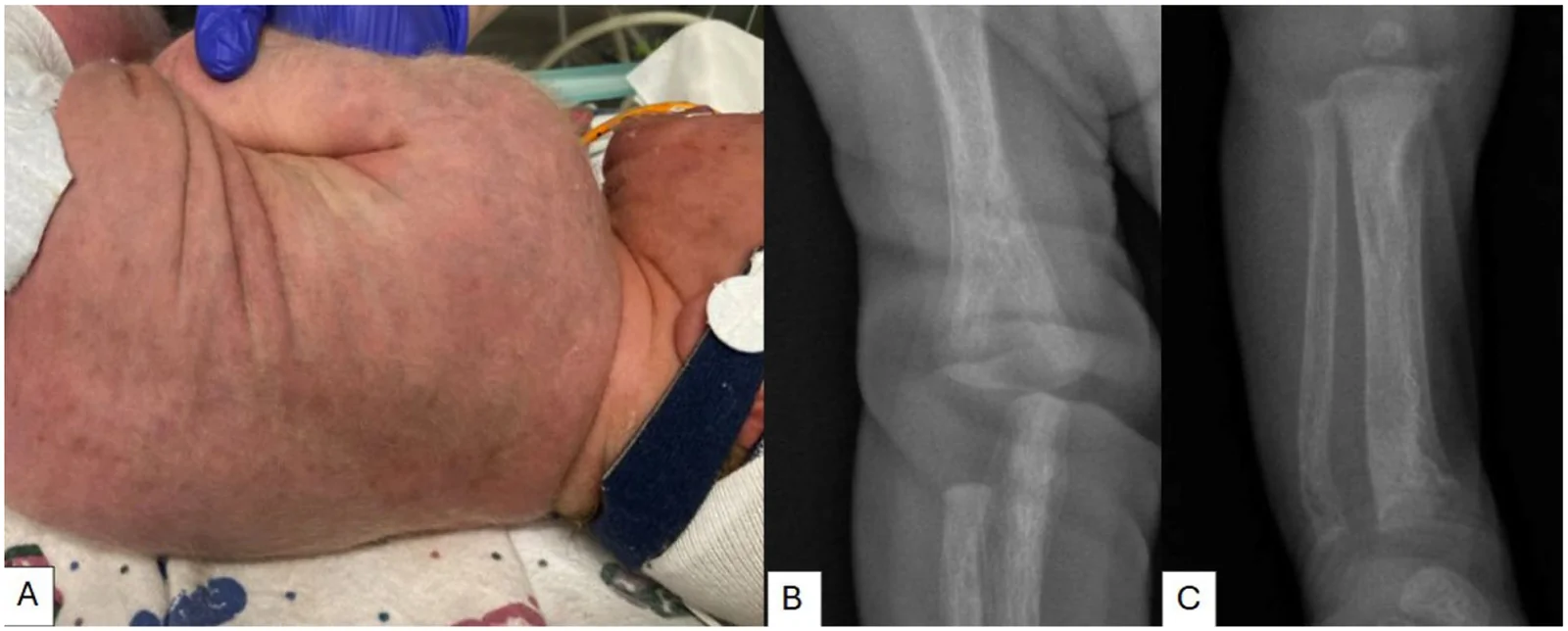
(A) Diffuse blueberry muffin rash on trunk and face noted at birth. (B) Widened diaphysis with diffuse diaphyseal cloaking and periosteal reactions. (C) Metaphyseal fragmentation prominent at the distal tibia and fibula which are medially angulated.

Additional laboratory findings were consistent with secondary hyperparathyroidism with persistently elevated alkaline phosphatase, elevated vitamin D 1,25, elevated parathyroid hormone, decreased phosphorus, and normal calcium.

The placenta was sent to the pathology lab for evaluation due to meconium staining and NICU admission. The placenta was received fresh and grossed according to institutional protocol. Photographs were taken of the fetal surface and umbilical cord. The placenta was grossed fresh. The umbilical cord was inserted eccentrically with slight twisting. The umbilical cord measured 65.4 cm in length with a diameter of 0.9 cm and was trivascular without significant abnormalities. 5 cm of umbilical cord was placed into formalin. The fetal surface showed a green discoloration. The membranes were complete with marginal insertion. A membrane roll was collected with the amnion side up and placed into formalin. The remaining cord and membrane were cut away leaving the placental disc. The placental disc measured 21.3 cm × 17.6 cm × 1.9 cm and weighed 605 g. A picture of the maternal surface was taken. The maternal surface was intact. The placenta was bread loafed with the maternal side up and showed red-brown cut surfaces. Three full thickness sections were taken: 1 from the cord insertion site and 2 from areas halfway between cord insertion and the disc margin. These sections were placed in formalin and fixed for 24 hours. Sections submitted for initial microscopic examination were a cross section of umbilical cord, 2 cross sections of membrane roll, placental disc at cord insertion site (full thickness), 2 sections of normal appearing placental disc (full thickness).

Microscopic evaluation of the membranes and umbilical cord were remarkable for the presence of meconium macrophages, chorioamnionitis, and funisitis. Microscopic examination of full thickness placenta disc sections were remarkable for diffuse vacuolation of syncytiotrophoblasts and villous stromal cells. Additional placental findings included mild decidual vasculopathy, intervillous thrombus with associated infarcted villi, normoblastemia, chronic villitis/intervillositis associated with increased perivillous fibrin and asvascular villi (confirmed with CD3 and CD68 IHC).

Upon initial microscopic examination, the extreme vacuolization of syncytiotrophoblasts and stromal villi led to suspicion that a specimen drop-off error or grossing error occurred. Investigation into the delivery of the specimen and grossing process confirmed that no errors occurred. Additional sections of fresh placenta were submitted for formalin fixation as per our grossing protocol to further address any processing errors. With the additional sections of placenta displaying consistent morphology, metabolic storage disorders were suspected. Further attempts to visualize the intracytoplasmic inclusions of the syncytiotrophoblasts with PAS, PAS-D, Alcian blue, and mucicarmine failed to stain the intracytoplasmic lipid inclusions. Oil Red-O was then considered as a potential stain to visualize intracytoplasmic inclusions. To utilize Oil Red-O fresh frozen placenta was required, which deviates from our standard protocol. Protocol deviation forms were completed and staining with Oil Red-O on fresh frozen placenta was performed (Figure 2). Muscle biopsy served as a positive control.

**Figure 2.**
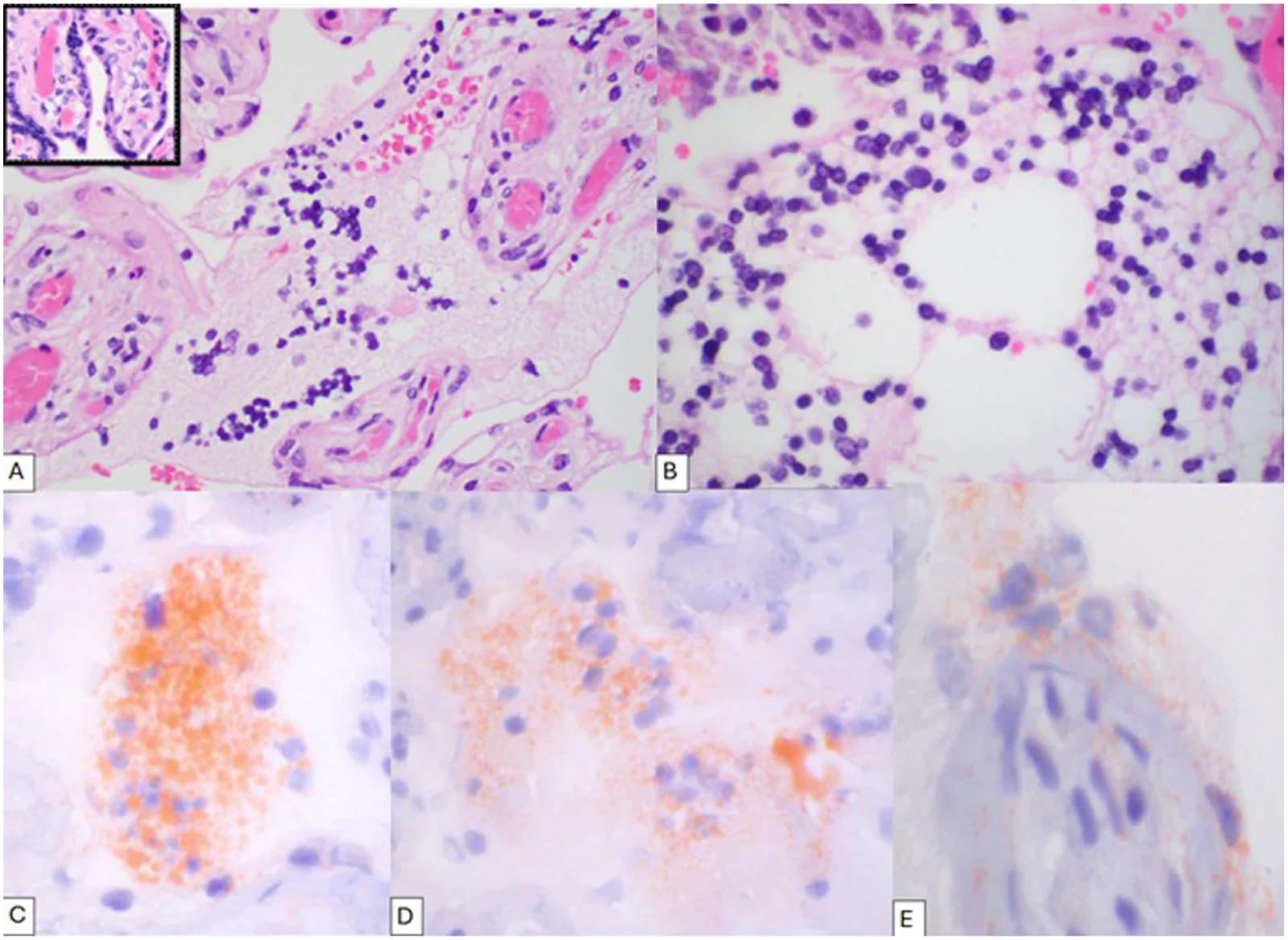
(A) High power (400x) H&E-stained section of the patient’s placenta disc showing diffuse vacuolation of syncytiotrophoblasts and villous stromal cells with normal placenta inset. (B) High power (600x) H&E-stained diffusely vacuolized syncytiotrophoblasts. (C and D) High power (600x) of Oil Red-O staining of innumerable intracytoplasmic inclusions of vacuolized syncytiorophoblasts. (E) High power (600x) of Oil Red-O staining of vacuolized syncytiotrophoblast and stromal villous cells.

The infant was transferred to level IV of the NICU for further evaluation. Ultra-rapid whole genome sequencing was performed with both the proband (patient) and paternal buccal samples. Two variants in the *GNPTAB* gene were identified; a pathogenic heterozygous missense variant in *GNPTAB* (c.2956C>T, p.Arg986Cys) known to be associated with reduced GlcNAc-1-phosphotransferase activity, and a likely pathogenic frameshift variant in *GNPTAB* (c.1833_1836del, p.His612LeufsTer17) known to be associated with loss of function through truncation or nonsense-mediated mRNA decay. Paternal sequencing confirmed the compound heterozygous nature of inheritance for these 2 pathologic *GNPTAB* variants. His urine oligosaccharide screen was positive and urine quantitative mucopolysaccharides resulted in high heparan sulfate (0.65 mg/mmol).

Based on his clinical presentation, urinary labs, placental histology, and molecular findings, he was diagnosed with I-cell disease/Mucolipidosis II. The family received genetic counseling, and the patient was discharged home at 11 days old with follow-up scheduled for multiple specialists, including Cardiology, Endocrinology, Genetics, Neurology, Ophthalmology, and Orthopedics.

Given the characteristic histologic findings observed on placental examination and the identification of compound heterozygous pathogenic *GNPTAB* variants identified by whole genome sequencing of the neonate, we next investigated whether sequencing of placental tissue could serve as an alternative approach for confirming the diagnosis. We selected a tissue block containing minimal decidua to minimize interference from maternal DNA and conducted whole exome sequencing on placental tissue. We confirmed the same pathogenic variants identified in the patient’s buccal sample—a; missense (c.2965C>T p.Arg986Cys) and frameshift variant (c.1833_1836del p.H612Lfs*17) in *GNPTAB*. Variant allele frequencies were approximately 50%, confirming heterozygous inheritance, although without paternal specimens compound heterozygosity could not be confirmed. Maternal DNA contamination was minimal and did not interfere with variant analysis or interpretation.

## Discussion

Mucolipidosis type II is caused by the loss of function of the GNPTAB gene. The absence of this gene leads to deficient uridine diphosphate-N-acetylglucosamine:lyosomal enzyme N-acetylglucosamine-1-phosphotransferase (G1cNAc-phosphotransferase) and defective lysosomal trafficking of lysosomal hydrolases from the trans-Golgi network to the lysosome due to lack of tagging with a mannose-6-phosphate. This leads to lysosomal hydrolases being secreted from the cell and accumulation of substrates (cholesterol, phospholipids, and glycosaminoglycans) in various tissues.^2,3^ Reduced activity of GNPTAB results in Mucolipidosis type III/ pseudo-Hurler polydystrophy, which presents similarly to Mucolipidosis type II but with later onset, slower progression and longer survival.^2^

The placenta is often under-appreciated in its diagnostic capacity. Histological examination of the placenta can reveal significant insight into the conditions of both the newborn and the mother.^9^ The pathologist examining the placenta should be suspicious for metabolic storage disorders in the setting of vacuolated syncytitrophoblasts and villous stromal cells. The substances that accumulate within the lysosomes in I-cell disease include cholesterol, phospholipids, and glycosaminoglycans. These accumulating substances can be detected in fresh frozen placenta using Oil Red-O special stain. To our knowledge, this is the first case to report successful staining of syncytiotrophoblast intracytoplasmic lipids using Oil Red-O special stain on fresh frozen placenta in the setting of I-cell disease.

Lipid content of placentas should be considered in the context of maternal obesity as in our case where maternal was BMI 34 kg/m^2^. Placentas of obese mothers have been shown to have higher lipid content than placentas of non-obese mothers.^10^ Studies that evaluated morphological differences between placentas of non-obese and obese mothers on light microscopy with H&E have found increased muscularity of placental vessels in obese mothers and a higher prevalence of placental immaturity in obese mothers.^11,12^ Regarding our case the degree of vacuolization of syncytiotrophoblasts and villous stromal cells seen on H&E is much more prominent than what would be expected from maternal obesity alone.

The placenta findings in this case are in line with other reported cases that discuss the manifestation of I-cell disease in the placenta. Grossly there was nothing remarkable about this placenta to indicate the presence of a metabolic storage disorder, which has been previously reported.^6,7^ The histological findings of previously reported cases include fine vacuolization of syncytiotrophoblasts and stromal expansion by vacuolized Hofbauer cells.^6-8^ Electron microscopy has been a longstanding method for ultrastructural examination of the placenta affected by I-cell disease.^5-7^ We proposed a special stain-based approach to visualize intracytoplasmic lipids with light microscopy. This method is by no means specific for the diagnosis of I-cell disease in the placenta but may be helpful in the work up of the rare case of a mucolipidosis disorder.

While the diagnosis of I-cell disease was ultimately solidified with rapid whole-genome sequencing of the infant, given the characteristic histologic findings of the placenta, we inquired whether placental tissue could be used for genetic diagnosis and confirmed its suitability. Pathologists are quite familiar with reflex molecular testing in the setting of malignancy—certain features or staining patterns trigger submission for molecular analysis of pathogenic variants. We anticipate that genetic testing of the placenta could represent a similar process. In addition to clinical, radiographic, biochemical, and genetic findings, we demonstrate here that placental histology and genetics can provide important diagnostic information in the setting of lysosomal disorders. One limitation of placental exome sequencing in this case is that we only analyzed the proband, and thus compound heterozygosity cannot be definitively determined. For diagnosis, placental sequencing would require type variant analysis of the parents to establish phase and potentially resolve maternal contamination if present. Taken together, these findings provide proof of concept for a pathology-directed diagnostic approach, highlighting that the placenta represents a rich source of material for both morphologic and genetic diagnosis. Antenatal diagnosis of I-cell disease has been previously reported via biochemical analysis of amniotic fluid and culturing of amniotic cells. In the modern molecular era our case raises the question; can molecular analysis of samples obtained by amniocentesis or chorionic villous sampling provide antenatal diagnosis of lysosomal storage disorders? Further cases are needed to corroborate our findings.
